# Acute Kidney Injury Following Posaconazole for Mucormycosis: SARS-CoV-2 as a Back-Seat Driver

**DOI:** 10.7759/cureus.27018

**Published:** 2022-07-19

**Authors:** Mohammad Noor, Said Amin, Fawad Rahim, Barkat Ali, Sheraz Zafar

**Affiliations:** 1 Internal Medicine, Khyber Girls Medical College, Peshawar, PAK; 2 Internal Medicine, Hayatabad Medical Complex Peshawar, Peshawar, PAK

**Keywords:** sars-cov-2 (severe acute respiratory syndrome coronavirus 2), fungal sinusitis, anti-fungal treatment, triazoles, thrombotic microangiopathy (tma), acute kidney injury, posaconazole, rhino-orbital mucormycosis, mucormycosis, covid-19

## Abstract

Viruses have been implicated in the causation of several systemic illnesses, either directly or by immune modulation. Severe acute respiratory syndrome coronavirus 2 (SARS-CoV-2) is not an exception. Due to altered immune regulation, it is often associated with novel clinical manifestations and complications which have not been reported before. SARS-CoV-2 induces a pro-inflammatory state which makes the patient vulnerable to developing a variety of previously unreported adverse reactions to medications. Coronavirus disease 2019 (COVID-19) and its treatment have provided a fertile ground for various opportunistic infections including mucormycosis. The standard treatment for mucormycosis is surgical debridement and liposomal amphotericin B. Triazole antifungals such as posaconazole and isavuconazonium are the second-line agents for those intolerant to first-line therapy. Posaconazole is safer than amphotericin B as far as renal adverse effects are concerned.

We report the case of a 60-year-old lady with type 2 diabetes mellitus, hypertension, ischemic heart disease, and osteoarthritis. She had severe COVID-19 requiring non-invasive ventilation four months ago. She presented with right rhino-orbital swelling, diplopia, and serosanguinous discharge from the right nostril. She had right third, sixth, and seventh cranial nerve palsies. Magnetic resonance imaging revealed right maxillary, ethmoid, and frontal sinusitis. Biopsy from the right nostril confirmed mucormycosis. Having normal renal and liver functions, she was started on oral posaconazole as she had an allergic reaction to a test dose of 1 mg amphotericin B (non-liposomal) in 20 mL of 5% dextrose water infused over 30 minutes. On day five, she developed acute kidney injury requiring renal replacement therapy. Her posaconazole was stopped. As she was not improving with conservative treatment, an ultrasound-guided, percutaneous renal biopsy was performed from the left kidney. The renal biopsy revealed thrombotic microangiopathy. She was started on liposomal amphotericin B as decided by the multidisciplinary team. Her renal function improved, and she continued on liposomal amphotericin B.

We conclude that thrombotic microangiopathy, in this case, was likely due to posaconazole. This is a novel adverse effect presumably of posaconazole. This case report will alert physicians to be vigilant of the renal adverse effects of posaconazole in patients who have had COVID-19. Patients who develop renal injury while on posaconazole should undergo an early renal biopsy to ascertain the exact histopathology.

## Introduction

Coronavirus disease 2019 (COVID-19) was initially thought to be a standalone respiratory illness [1]. As the pandemic is evolving, it has unraveled a variety of systemic complications. The systemic complications are related to respiratory, cardiovascular, gastrointestinal, hematological, rheumatological, cutaneous, neurological, and renal systems [2]. These are either due to direct infection by severe acute respiratory syndrome coronavirus 2 (SARS-CoV-2), immune modulation by the virus, or due to the drugs used to treat COVID-19. Viral infections have been reported to induce a pro-inflammatory state in the body which predisposes the patient to a variety of previously unreported adverse reactions to medications [3]. Apart from the immune modulation by SARS-CoV-2, steroids, tocilizumab, and other immunosuppressants used for the treatment of COVID-19 contributed to the emergence of opportunistic infections such as mucormycosis [4].

Mucormycosis used to be a rare disorder, accounting for 0.005 to 1.7 cases per million population per year worldwide [5]. The prevalence of mucormycosis has increased, and up to 140 cases per million population were reported in India during the COVID-19 pandemic [5]. Surgical debridement and liposomal amphotericin B are the standard initial treatment options. In Pakistan, non-liposomal amphotericin B is still used because of financial constraints and the non-availability of liposomal amphotericin B. Posaconazole and isavuconazole are the second-line treatment options for patients who are intolerant to amphotericin B [6]. The most commonly reported adverse effects of posaconazole treatment are gastrointestinal including nausea, vomiting, abdominal pain, elevated liver enzymes, headache, rash, prolongation of QT interval, and neuropathies [7]. Renal adverse effects of posaconazole are rare and occur only in 0.1-1% of patients [8]. Only one case of acute kidney injury (AKI) caused by posaconazole has been reported where a renal biopsy was not performed, and the renal function improved after stopping posaconazole [9].

We report the case of a 60-year-old female with diabetes mellitus (DM), hypertension (HTN), ischemic heart disease (IHD), osteoarthritis (OA), post-COVID-19, and rhino-orbital mucormycosis. She was initially treated with surgical debridement and non-liposomal amphotericin which was stopped due to an allergic reaction. She was started on oral posaconazole 300 mg daily. On day five, she developed AKI requiring renal replacement therapy. She remained dialysis-dependent and had an ultrasound-guided percutaneous renal biopsy on day 14. The renal histology revealed thrombotic angiopathy. Her posaconazole was stopped. Her renal functions improved after stopping posaconazole. Special arrangements were made for liposomal amphotericin B. She was started on intravenous liposomal amphotericin B.

## Case presentation

A 60-year-old female presented with right-sided headaches and swelling of the right cheek and the right orbital region for the last 10 days. The pain and swelling started gradually with low-grade fever. She complained of double vision and difficulty in opening and closing her right eye, but her vision was normal. She had difficulty swallowing as food material would stay on the right side of her oral cavity. She complained of a blocked nose, unpleasant smell, and serosanguinous discharge from the right nostril. There was no history of morning stiffness and jaw claudication.

She had DM for the last 16 years with poor glycemic control. In addition, she had HTN and IHD for the last six years. Three months ago, she had acute anterior wall myocardial infarction and underwent thrombolysis. She was hospitalized for severe COVID-19 two months back. She received standard care along with intravenous dexamethasone 6 mg daily for 10 days and non-invasive ventilation for two weeks. She made an uneventful recovery from COVID-19 and was advised to continue treatment for DM, HTN, and IHD. She was taking aspirin, rosuvastatin, ramipril, bisoprolol, furosemide, empagliflozin, metformin, omeprazole, and glargine insulin. She used to take paracetamol for OA of the knees as needed.

**Figure 1:**
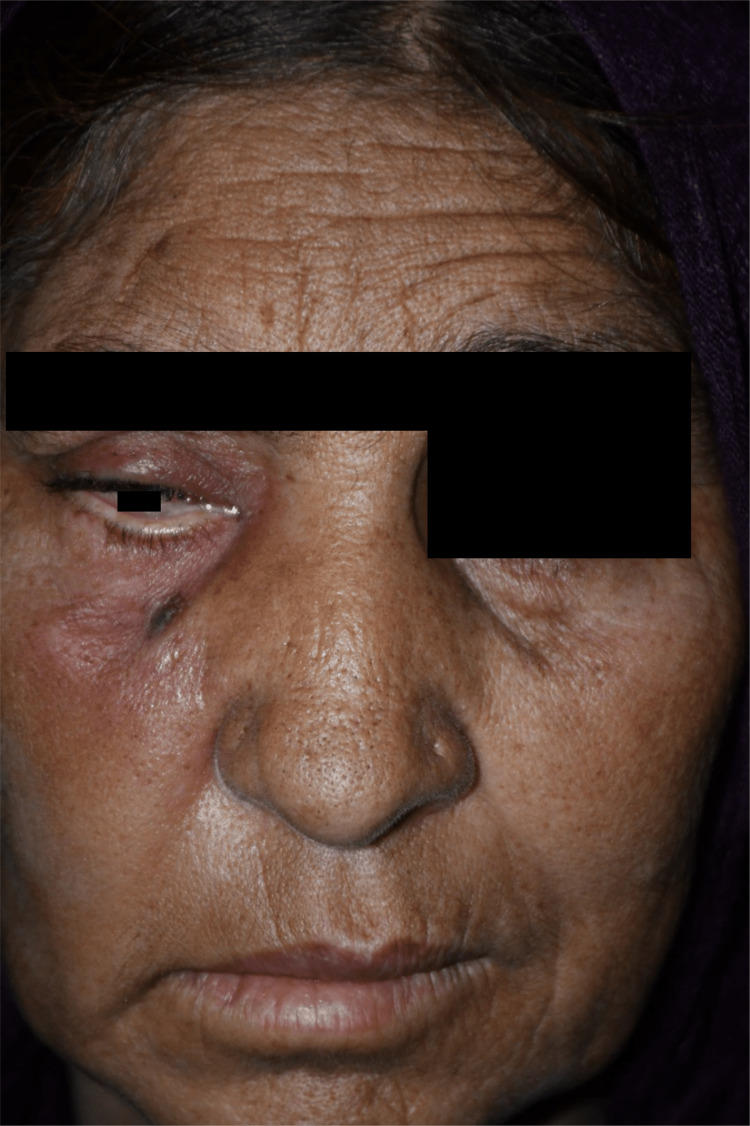
Photograph of the patient at the time of admission showing swelling of the right cheek, right-sided ptosis, and right-sided lower motor neuron facial nerve palsy.

She received oral co-amoxiclav from her general practitioner for suspected sinusitis without any improvement. She was referred to Hayatabad Medical Complex, Peshawar, Pakistan for management. She was admitted for further workup. Examination revealed a pulse of 96 beats per minute, a temperature of 100°F, a blood pressure of 150/100 mmHg, oxygen saturation of 95% on room air, and weight of 75 kg. Her Glasgow Coma Scale (GCS) score was 15/15. She had a tender and swollen right cheek with drooping of the right eyelid with loss of wrinkles on the right half of the forehead. The right eye had restricted movements, and there was paralysis of the right third, sixth and seventh cranial nerves. She had a dilated right pupil, and her visual acuity was 6/12 in both eyes. Fundus examination showed background diabetic and hypertensive retinopathy. The right side of the nose was swollen, and there was a serosanguinous discharge and blackish crust in the right nostril (Figure 1).

The rest of the neurological and systemic examination was unremarkable. Her investigations at the time of admission are summarized in Table 1.

**Table 1:** Investigations at the time of admission to the tertiary care hospital. HbA1C: glycosylated hemoglobin; CRP: C-reactive protein; ELISA: enzyme-linked immunosorbent assay; HBsAg: hepatitis B surface antigen; HCV: hepatitis C virus; HIV: human immunodeficiency virus; SARS-CoV-2: severe acute respiratory syndrome coronavirus 2; PCR: polymerase chain reaction; PA: posterior-anterior

Investigations	Reference range	Results
Hemoglobin (g/dL)	11.5–17.5	11.2
Platelets counts (×10^3^/µL)	150–450	546
White cell count (×10/µL)	4–11	13.35
Neutrophils (%)	40–75	73
Lymphocytes (%)	20–45	16
Monocytes (%)-	2–10	06
Eosinophil (%)	1–8	05
Blood urea (mg/dl)	18–45	45
Serum creatinine (mg/dL)	0.2–1.2	1.1
Total bilirubin (mg/dL)	0.1–1	0.2
Alanine aminotransferase (IU/L)	10–50	30
Alkaline phosphatase (IU/L)	35–104	134
Blood glucose (random) (mg/dL)	70–140	196
HbA1C (%)	<5.7 %	10.6
Serum sodium (mEq/L)	135–145	139
Serum potassium (mEq/L)	3.5–5.5	3.8
Serum chloride (mEq/L)	96–112	104
CRP (mg/dL)	<0.5	4.6
HBsAg (ELISA)	Non-reactive	Non-reactive
Anti-HCV (ELISA)	Non-reactive	Non-reactive
Anti-HIV (ELISA)	Non-reactive	Non-reactive
SARS-CoV-2 PCR	Negative	Negative
Urinalysis	+ Glucose	
Chest X-ray	Normal	
Transthoracic echocardiography	Normal	
Ultrasound of abdomen and pelvis	Fatty liver, otherwise normal	

**Figure 2:**
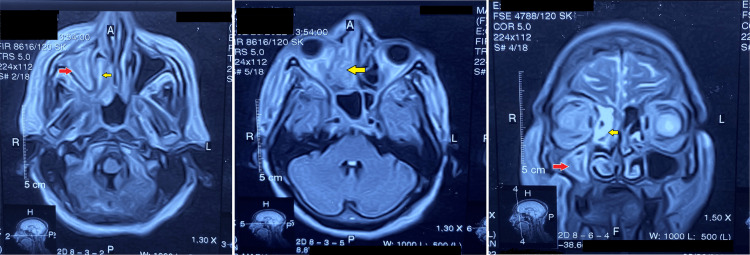
Magnetic resonance imaging of paranasal sinuses (axial and coronal sections) showing thickening of the right maxillary sinus mucosa (red arrows) and right ethmoid air cells (yellow arrows) with internal hypointense linear components suggestive of fungal sinusitis.

Differential diagnoses of mucormycosis, complicated bacterial sinusitis, orbital cellulitis, dental abscess, orbital and nasopharyngeal tumors, and granulomatosis with polyangiitis (GPA) were considered in the multidisciplinary team (MDT) meeting. She underwent magnetic resonance imaging (MRI) of the paranasal sinuses (Figure 2).

She was booked for examination under anesthesia and biopsy from the nasal cavity and paranasal sinuses. Examination under anesthesia revealed swollen mucosa of the right nostril, blackish crust, and serosanguinous discharge from the paranasal sinuses. Surgical debridement was done, and a biopsy was sent for histopathology and fungal staining. Due to the non-availability of liposomal amphotericin B, she was given a test dose of 1 mg amphotericin B (non-liposomal) in 20 mL of 5% dextrose water infused over 30 minutes. She had an allergic reaction to the test dose and the infusion was stopped. Based on the decision during the MDT meeting, she was started on oral posaconazole 300 mg daily. She developed anorexia, vomiting, and oliguria. Her renal functions revealed AKI, and schistocytes were reported in the peripheral smear. The posaconazole was stopped, and conservative management was instituted for the AKI. She developed a fluid overload state with azotemia and preserved ejection fraction. Her renal functions further deteriorated necessitating renal replacement therapy.

She was offered hemodialysis for two weeks. Despite two weeks of hemodialysis and conservative treatment, there was no sign of any improvement in the renal functions. The MDT decided to proceed with an ultrasound-guided percutaneous renal biopsy to determine the exact nature of the renal pathology. She underwent an uneventful percutaneous biopsy from the left kidney. She was dialyzed for a further four weeks when gradually her renal functions normalized. Her platelet count and reticulocyte count remained normal; however, her D-dimer levels were 1.2 µg/mL (normal range = <0.5 µg/mL). Her renal functions and hemoglobin level over the course of her stay are summarized in Table 2.

**Table 2:** Trend of renal functions and hemoglobin over the course of hospital stay and at the first follow-up visit.

Investigations	Reference range	Days since admission										
		01	05	10	15	20	25	30	40	50	60	Follow-up visit
Hemoglobin (g/dL)	11.5–17.5	11.2	7.8	8.2	7.7	7.6	8.4	9.0	9.7	10.4	11.2	11.0
Blood urea (mg/dL)	18–45	45	140	78	96	148	95	81	53	55	49	42
Serum creatinine (mg/dL)	0.2–1.2	1.1	6.1	2	2.9	4.2	2.4	2.2	1.2	1.1	1.1	1.2
Serum sodium (mEq/L)	135–145	139	134	132	125	132	122	128	123	132	130	134
Serum Potassium (mEq/L)	3.5–5.5	3.8	5.6	4.5	4.6	5.5	5.3	4.1	3.5	3.2	3.4	3.6
Serum chloride (mEq/L)	96–112	104	101	100.5	86	107	87	88.7	84.7	87.8	90.7	94

**Figure 3:**
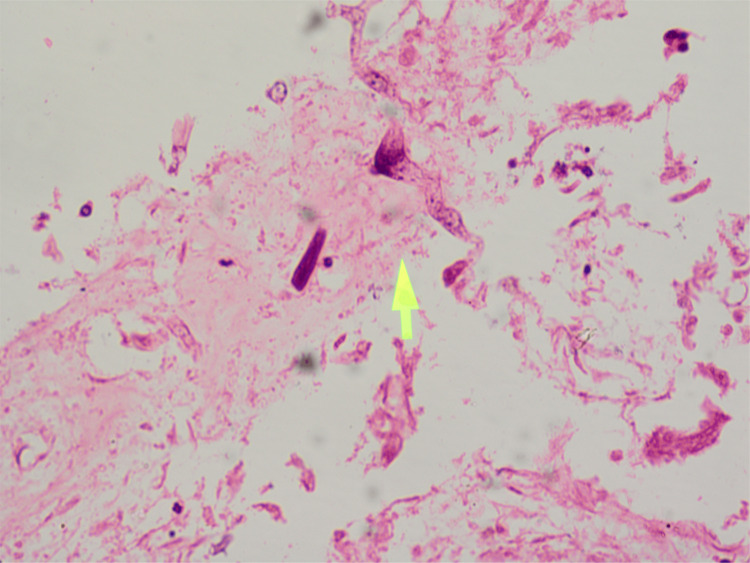
Biopsy of the nasal mucosa (hematoxylin and eosin stain) showing septate hyphae of Mucor.

**Figure 4:**
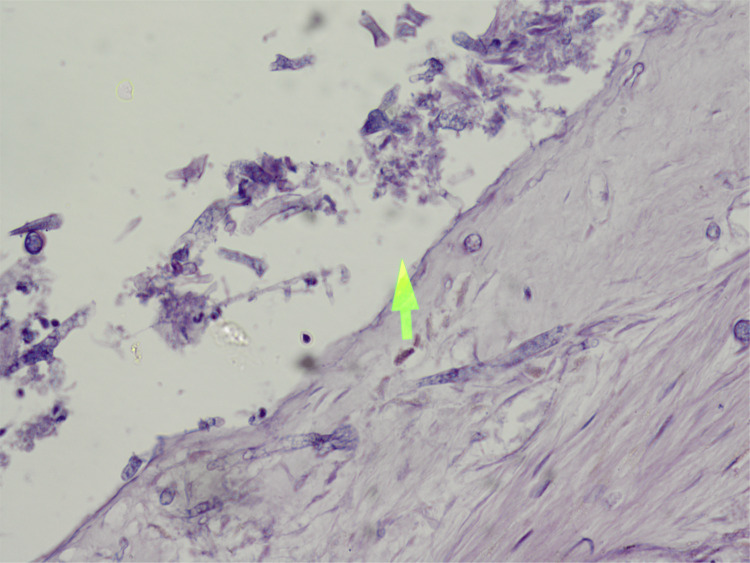
Biopsy of the nasal mucosa (Periodic acid-Schiff stain) showing septate hyphae of Mucor.

**Figure 5:**
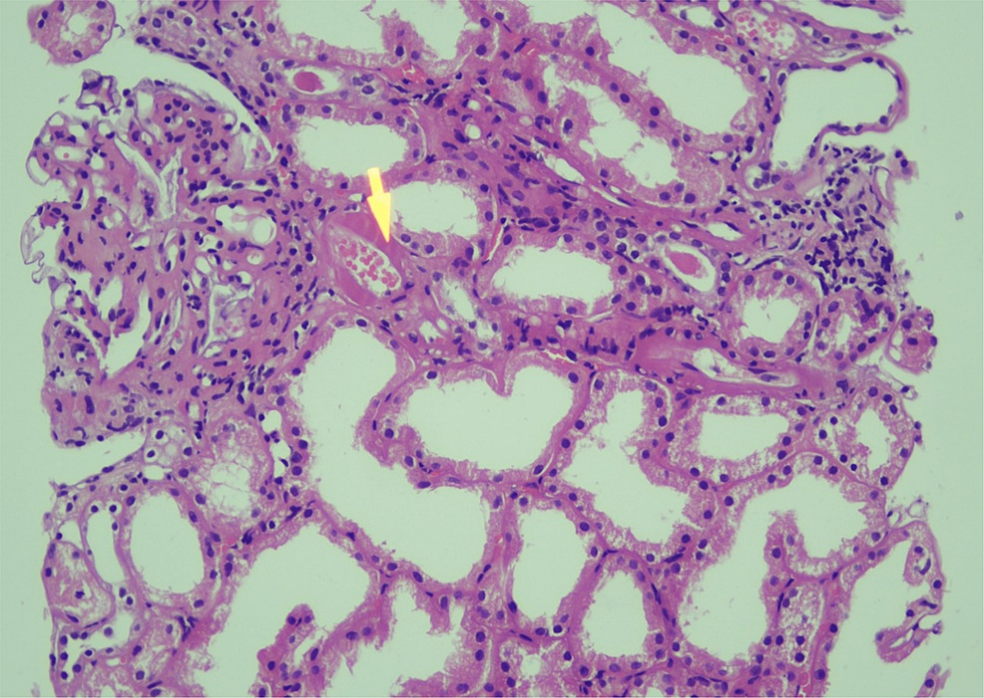
Renal biopsy (hematoxylin and eosin stain, medium-power view) showing a renal vessel with fibrinoid necrosis of its wall (pink arrow).

**Figure 6:**
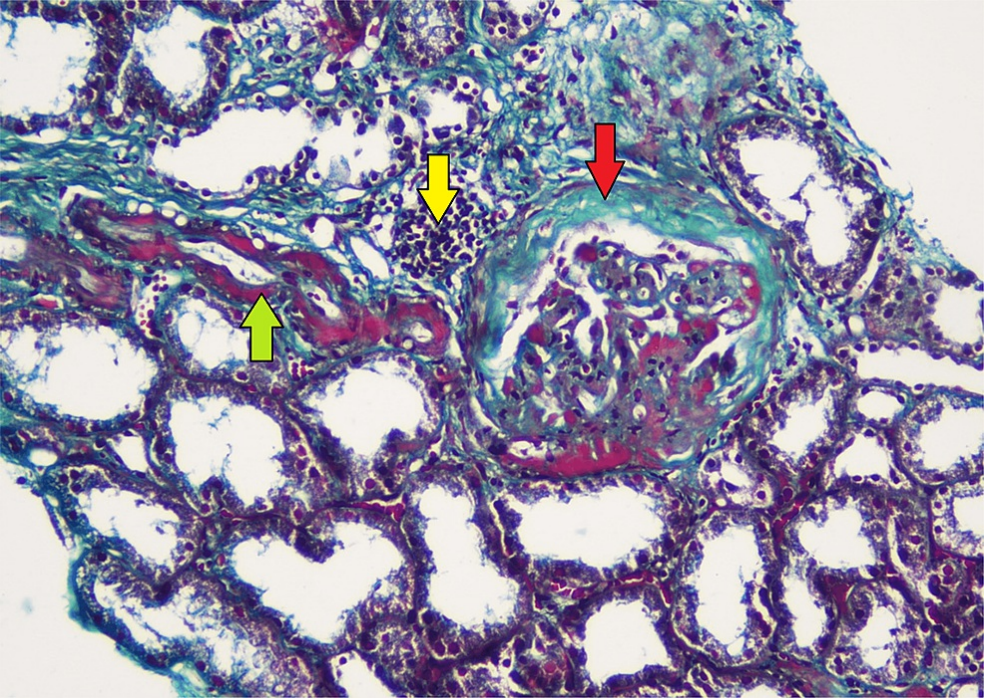
Renal biopsy (trichome stain, medium-power view) showing periglomerular thickening (red arrow), interstitial inflammation (yellow arrow), and thickening of the small vessels (green arrow).

**Figure 7:**
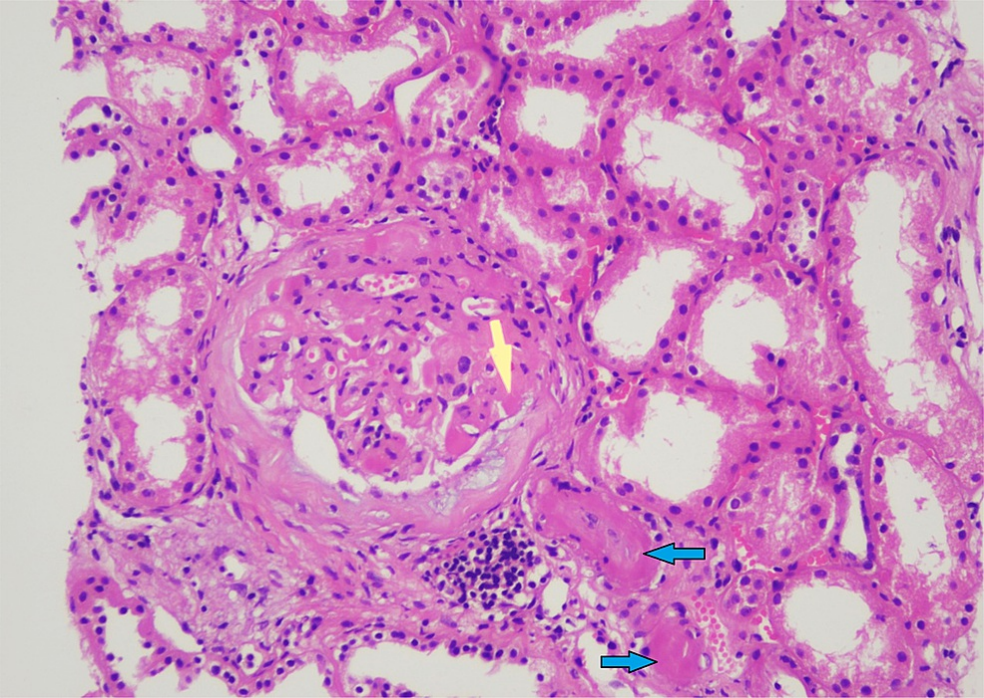
Renal biopsy (hematoxylin and eosin stain, high-power view) showing fibrinoid necrosis within glomerular capillaries (pink arrow) and interstitial vessels (blue arrows).

**Figure 8:**
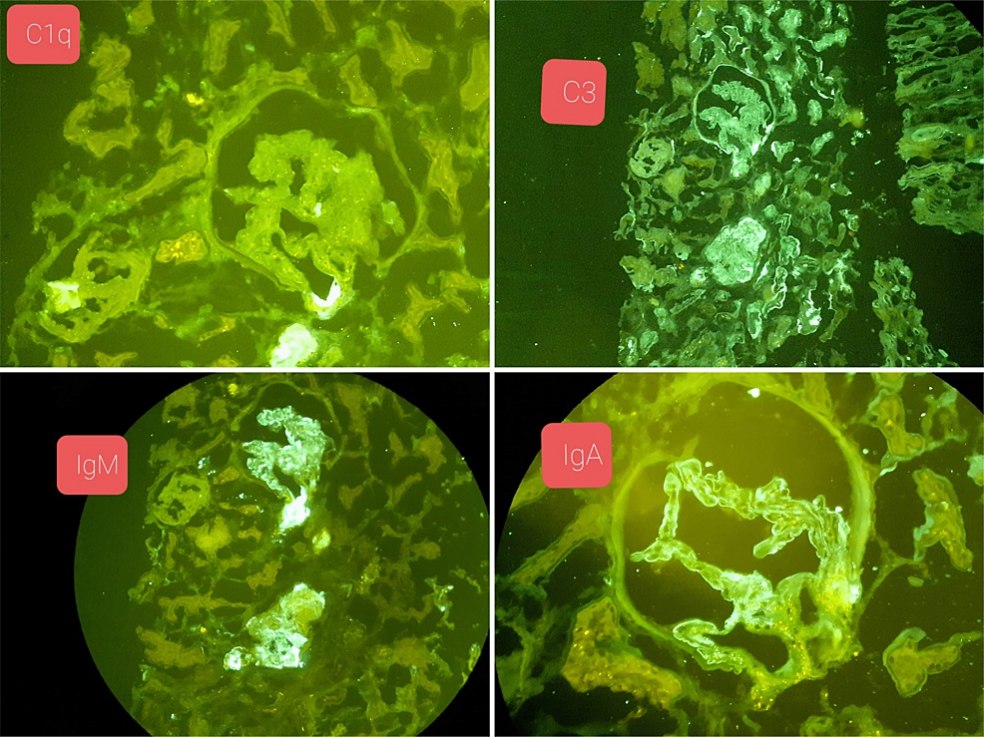
Renal biopsy (immunofluorescence) showing non-specific staining for C1, C3, IgM, and IgA. Ig: immunoglobulin

The biopsy from the nasal mucosa and the renal biopsy revealed mucormycosis and thrombotic microangiopathy, respectively (Figures 3-8).

**Figure 9:**
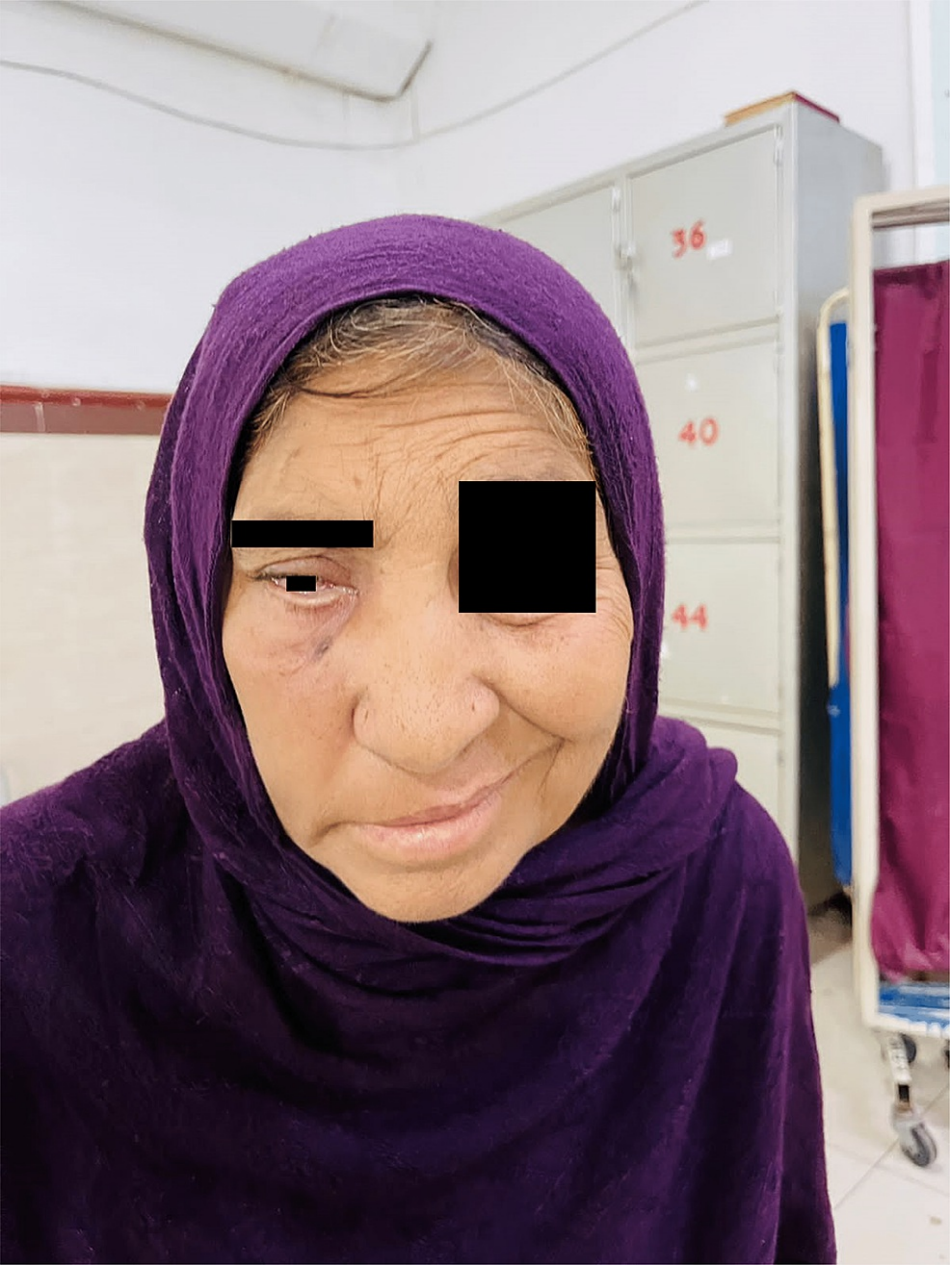
Photograph of the patient showing minimal swelling of the right cheek, right-sided ptosis, and right lower motor neuron facial nerve palsy.

Given her deteriorating condition and the wish of the patient and her family, the MDT decided to give her liposomal amphotericin B with extreme caution. Special arrangements were made for the provision of liposomal amphotericin B. She was started on liposomal amphotericin B (3 mg/kg/day). Her renal functions and electrolytes stayed within the normal range while she was on liposomal amphotericin B. Her rhino-orbital swelling resolved, but she had residual palsies of the right third, sixth, and seventh cranial nerves. She was discharged after two months of prolonged hospitalization. She was advised to continue liposomal amphotericin B at the local healthcare facility. She was advised to continue her medications and avoid nephrotoxic drugs and was booked for a follow-up in a month. She was reviewed in the outpatient clinic a month after discharge. There was minimal rhino-orbital swelling, and no nasal crust or discharge (Figure 9).

**Figure 10:**
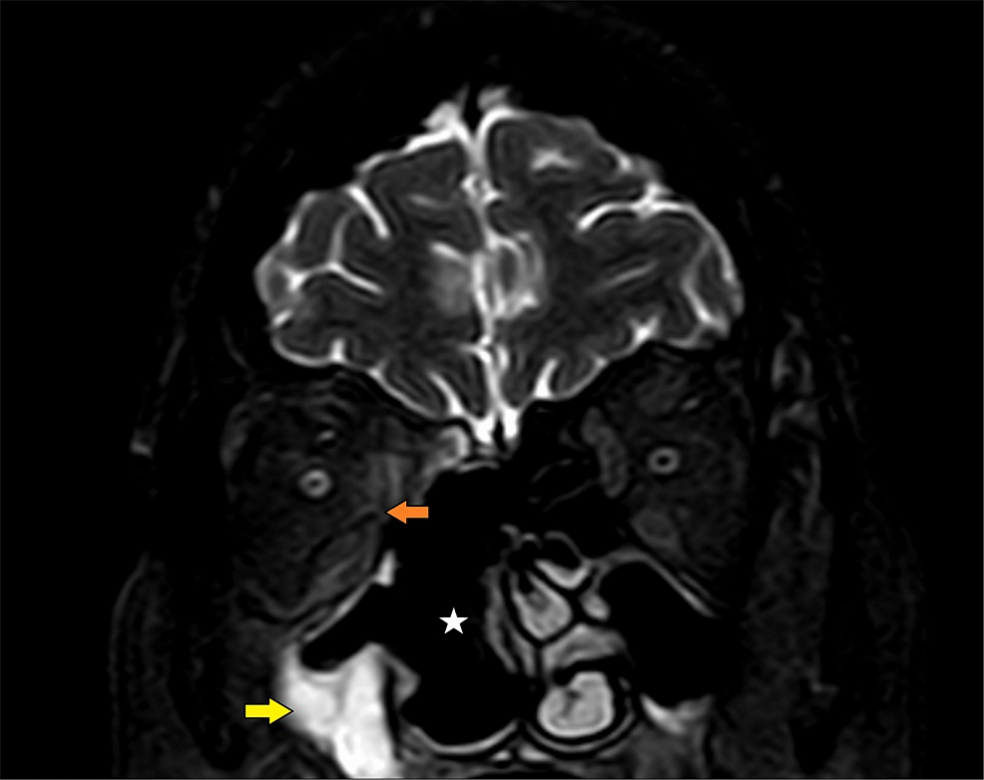
Magnetic resonance imaging (T2 fat-saturated coronal section) showing fluid and mucosal thickening in the right maxillary sinus (yellow arrow), high signals in the inferomedial quadrant of the right orbit (pink arrow), and non-visualization of nasal turbinates on the right side (white star) - postsurgical. Findings are suggestive of the extension of the disease into the orbit.

**Figure 11:**
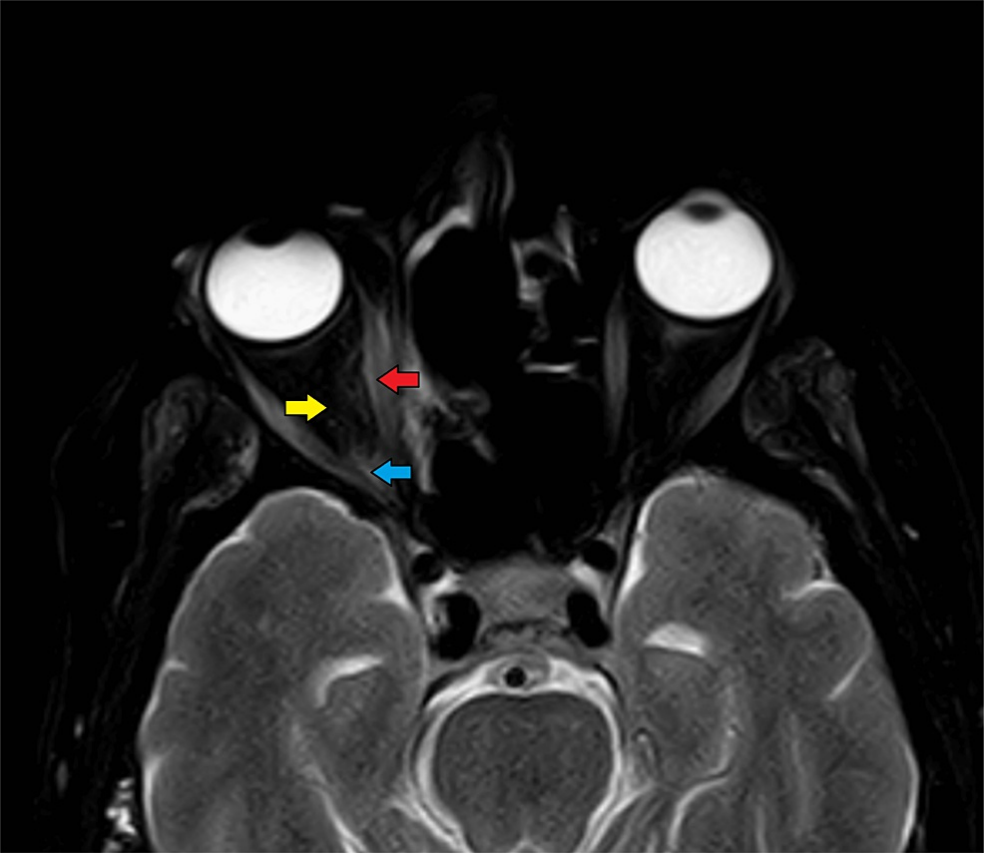
Magnetic resonance imaging (T2 fat-saturated axial section) showing retro-orbital fat stranding (yellow arrow), slightly bulky right medial rectus muscle (red arrow), and the extension of the disease process into the orbital apex (blue arrow).

**Figure 12:**
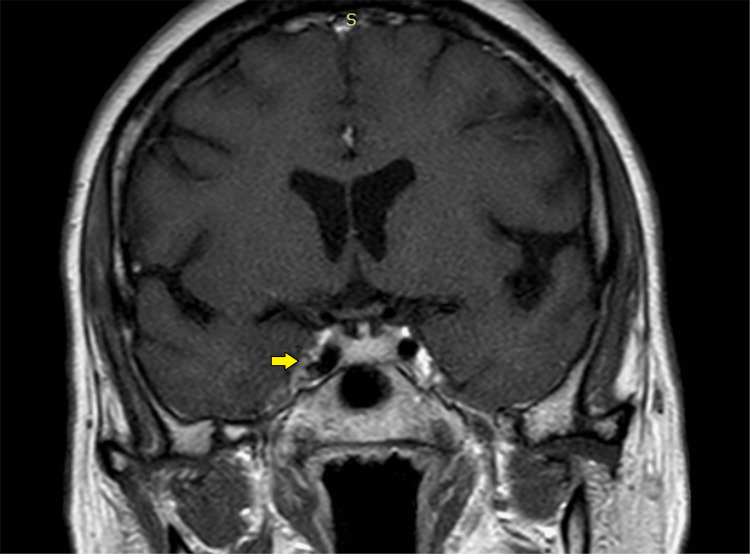
Magnetic resonance imaging (T1 post-contrast coronal section) showing focal involvement of the right cavernous sinus (yellow arrow) by the disease process.

**Figure 13:**
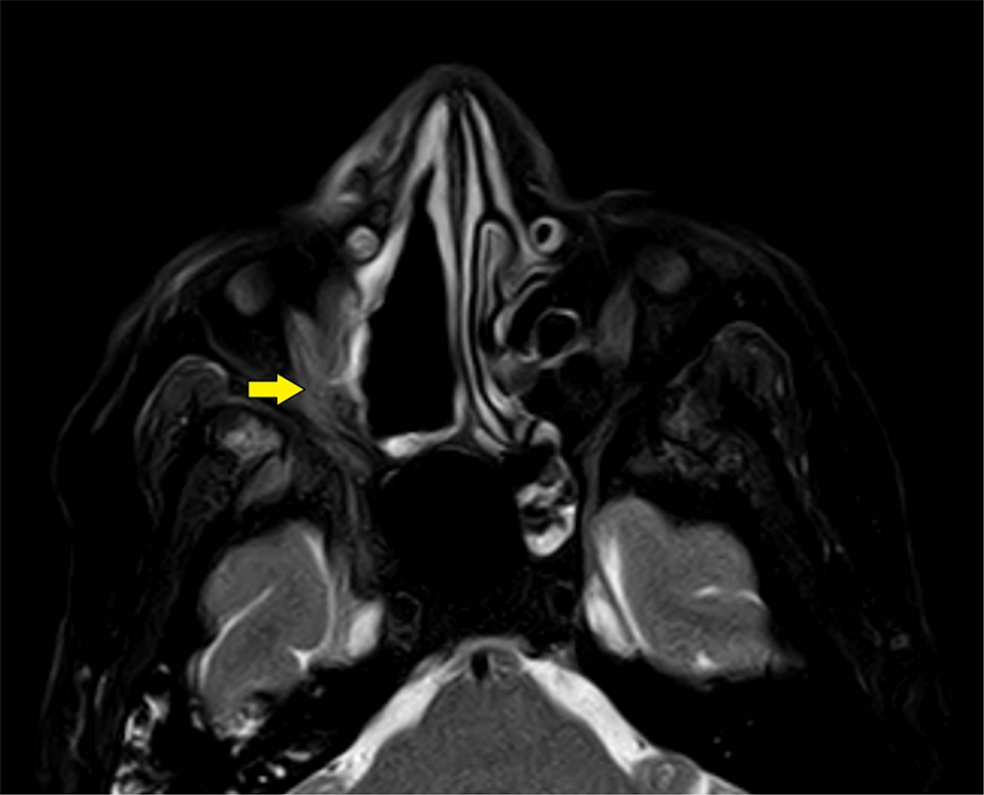
Magnetic resonance imaging (T2 fat-saturated axial section) showing obliteration of the retro-antral fat on the right side with high signals in the surrounding tissues (arrow) suggestive of disease extension into the infratemporal fossa.

**Figure 14:**
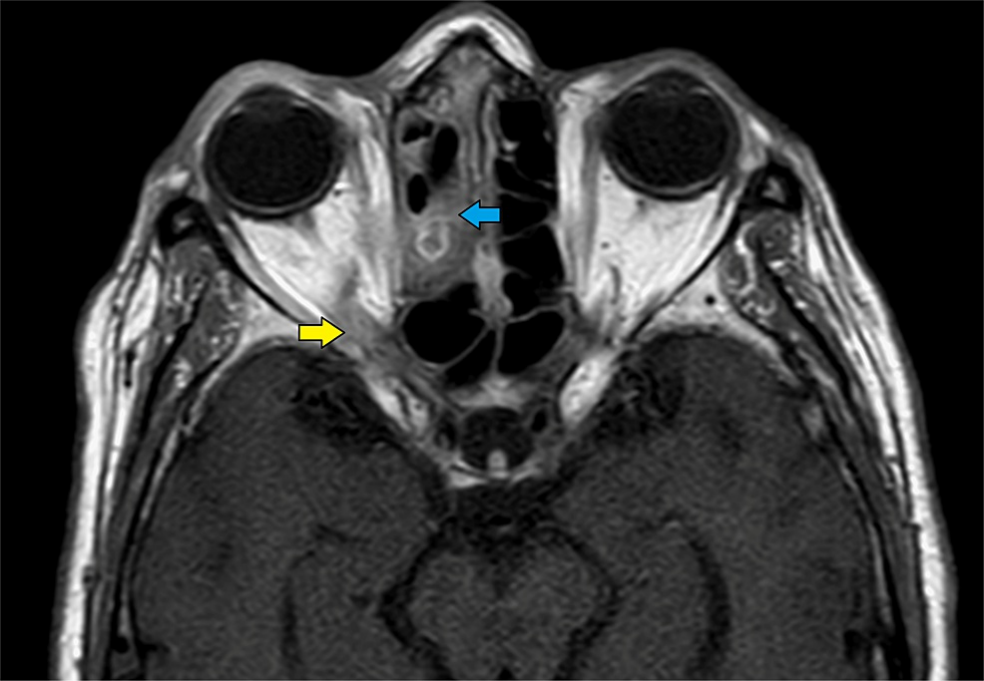
Magnetic resonance imaging (T1 post-contrast axial section) showing heterogeneous signals in the right orbital apex with focal extension into the cavernous sinus (yellow arrow) and heterogeneous enhancement of the mucosa along with fluid in the right ethmoid air cells (blue arrow).

Her renal functions remained within normal limits. However, a repeat MRI revealed residual disease in the sinuses and orbit (Figures 10-14).

The MDT decided to continue liposomal amphotericin B for another month at the local healthcare facility. She was booked to be reviewed in this hospital after a month.

## Discussion

Decision-making about patients with multiple comorbid conditions and starting treatment with drugs having a potential adverse effects profile can be a daunting task because of medical complexity, uncertainty about prognosis and treatment effectiveness, and setting priorities. The scenario gets more challenging for internists in developing countries with limited resources. The situation is further compounded by illiteracy and multiple family stakeholders who may need to be involved in decision-making [10].

Mucormycosis is also called malignant fungus as it is not only osteo-invasive and vaso-invasive but also disseminates just like a malignancy [11]. Deciding on the treatment of post-COVID-19 mucormycosis in frail patients with multiple comorbid conditions is of great concern to physicians as treatment with amphotericin-B and posaconazole is not without unfavorable adverse effects. Moreover, its treatment can be counterproductive and sometimes results in a great deal of morbidity and mortality [12].

The patient had DM, HTN, IHD, and recent severe COVID-19 requiring non-invasive ventilation, steroids, and antibiotics for two weeks, and was later discharged in a stable state. However, she presented four months later with right-sided painful rhino-orbital swelling. Diagnosis of mucormycosis was confirmed on radiological findings and tissue biopsy. She developed a severe allergic reaction soon after receiving the test dose of amphotericin B (non-liposomal). Based on the decision of the MDT meeting, she was started on a second-line antifungal, posaconazole. She developed AKI with posaconazole and underwent renal replacement therapy. Renal biopsy revealed thrombotic microangiopathy. Thrombotic microangiopathy in the renal vasculature has neither been reported with DM, HTN, IHD, mucormycosis nor with the medications that she was taking. Thrombotic microangiopathy and thrombotic thrombocytopenic purpura have been reported with acute COVID-19 illness, and following Ad26.COV2.S COVID-19 vaccine [13,14]. However, she had an uneventful recovery from COVID-19 four months ago. She had normal renal functions at the time of admission. Posaconazole has a relatively safe renal side effects profile. Only one case of AKI has been reported with posaconazole so far [9]. The histology was not shared as a renal biopsy was not performed, and the renal functions of the patient improved after stopping posaconazole.

We conclude that the thrombotic microangiopathy, in this case, was likely due to posaconazole which has not been reported in the literature before. Viral infections, particularly SARS-CoV-2, provoke a proinflammatory state in the body and predispose the patient to a variety of novel adverse reactions. Grover et al. have reported a case of toxic epidermolysis associated with tamsulosin after COVID-19 [3].

This case would add to the list of possible adverse effects of posaconazole and would help internists to consider the renal adverse effects of posaconazole. Regular monitoring of renal function is necessary for patients on posaconazole for the early identification of AKI. Moreover, patients who develop unexplained AKI following treatment with posaconazole should undergo a renal biopsy to determine the exact spectrum of renal histology. This becomes more significant in patients with altered immune status following COVID-19.

This case report has some limitations. The facilities to determine serum posaconazole level and the assay for a disintegrin and metalloproteinase with thrombospondin type 1 motifs, member 13 (ADAMTS13) were not available locally. The case report is not meant to establish a cause-and-effect relationship between posaconazole and thrombotic microangiopathy for which case series, case-control studies, and cohort studies are needed.

## Conclusions

Because of altered immune regulation, SARS-CoV-2 causes novel clinical presentations and adverse drug reactions. In patients who have had COVID-19, physicians should be aware of the rare renal adverse effects of posaconazole therapy. Thrombotic microangiopathy in association with posaconazole has not been reported in the literature. In patients with a history of COVID-19, early renal biopsy shall be considered if they develop AKI while being treated with posaconazole.
